# Awake craniotomy in patients with arteriovenous malformation: A systematic review and meta‑analysis

**DOI:** 10.3892/mi.2024.166

**Published:** 2024-06-06

**Authors:** Arya Harikrishna, Stefanos Chatzidakis, Angela Ishak, Konstantinos Faropoulos, George Fotakopoulos, Vasiliki Epameinondas Georgakopoulou, Pagona Sklapani, Nikolaos Trakas, Andreas Yiallouris, Christina Iosif, Aris P. Agouridis, George Hadjigeorgiou

**Affiliations:** 1 School of Medicine, European University Cyprus, 2404 Nicosia, Cyprus; 2 Department of Neurosurgery, Nicosia General Hospital, 2031 Nicosia, Cyprus; 3 Department of Neurosurgery, General University Hospital of Larissa, 41221 Larissa, Greece; 4 Department of Pathophysiology, National and Kapodistrian University of Athens, 11527 Athens, Greece; 5 Department of Biochemistry, Sismanogleio Hospital, 15126 Athens, Greece; 6 Department of Minimally Invasive Neurosurgery, Athens Medical Center, 14562 Athens, Greece; 7 Department of Neurosurgery, Apollonion Private Hospital, 2054 Nicosia, Cyprus

**Keywords:** arteriovenous malformations, awake craniotomy, neurologic outcomes, surgical resection

## Abstract

The present systematic review aimed to identify all the available literature on awake craniotomy (AC) in patients with arteriovenous malformation (AVM) in order to evaluate its safety, risks, benefits and effectiveness. All available literature on AC in patients with AVM was collected and evaluated in an aim to provide a better understanding of its safety, associated risks and benefits. A systematic search for studies employing AC in patients with AVM was conducted using the PubMed, Scopus and ScienceDirect databases without restrictions on the year of publication, language, or study design, from inception up to May 30, 2021. A total of 11 studies published between 2004 and 2021 with 106 patients who underwent ACs were considered eligible. The rate of complete resection was 93% [95% confidence interval (CI), 82 to 100%; I^2^ 0%]. The intraoperative complication rate was 21% (95% CI, 1 to 41%; I^2^ 55%) and the post-operative complication rate was 33% (95% CI, 19 to 48%; I^2^ 40%). During follow-up, the complication rate was 6% (95% CI, 1 to 10%; I^2^ 30%). The post-operative complication rate was higher in the Spetzler-Martin grade (SMG) III-V group (31%; 95% CI, 21 to 42%; I^2^ 46%) than in the SMG I-II group (12%; 95% CI, 2 to 22%; I^2^ 0%). Similarly, the follow-up complication rate was higher in the SMG III-V group (9%; 95% CI, 2 to 16%; I^2^ 34%) than in the SMG I-II group (0%; 95% CI, 0 to 4%; I^2^ 0%). On the whole, the present study provides preliminary evidence to indicate that AC is a possible and useful option for the resection of AVM in selected patients. Well-designed future studies with long-term follow-up are required however, to investigate various aspects of safety and provide solid data for AC in patients with AVM.

## Introduction

Cerebral vascular malformations present a range of lesions, among which arteriovenous malformation (AVM) is the most common (1). The management of AVM involves a multi-disciplinary approach and often requires a combination of treatments, including surgery, embolization and radiosurgery (RS) (2,3). The surgical removal of an arteriovenous malformation (AVM) can be challenging for any neurosurgeon (1,2). Depending on an the anatomic complexity, size and position of AVMs with regard to eloquent areas, a long surgical or a multidisciplinary team and numerous sessions may be required to achieve maximum resection (3). More specifically, the surgical maneuvers required for the resection of an AVM could jeopardize the neurological integrity of the patient, particularly if it is located in an eloquent area (4,5). AVMs are highly variable in size, shape and location. However, patterns emerge and subtypes with definable anatomy appear, enabling a certain degree of classification (3). The Spetzler-Martin grading (SMG) scale is the most commonly used classification method to describe AVMs. It considers the size of the AVM, the location of the nidus and the pattern of venous drainage (3). The development of brain mapping techniques, such as functional magnetic resonance imaging (fMRI), electrocorticography (ECoG), intracranial electroencephalography and electrical stimulation during awake craniotomy (AC) could aid in the modern neurosurgical treatment of AVMs in eloquent areas (3,6). As for the first choice, there is evidence to suggest that fMRI may not be helpful during the resection of AVMs in eloquent areas (6). To the best of our knowledge, this is the first meta-analysis to evaluate the role of AC in patients with AVM. The main aim of the present study was to assess the associated risks and benefits of AC in the resection of AVMs by recording all the neurological outcomes. The present also aimed to identify the rate of complete resection, intraoperative and post-operative complications, and subgroups of individuals who will benefit from AC.

## Data and methods

### Search strategy

**Figure 1:**
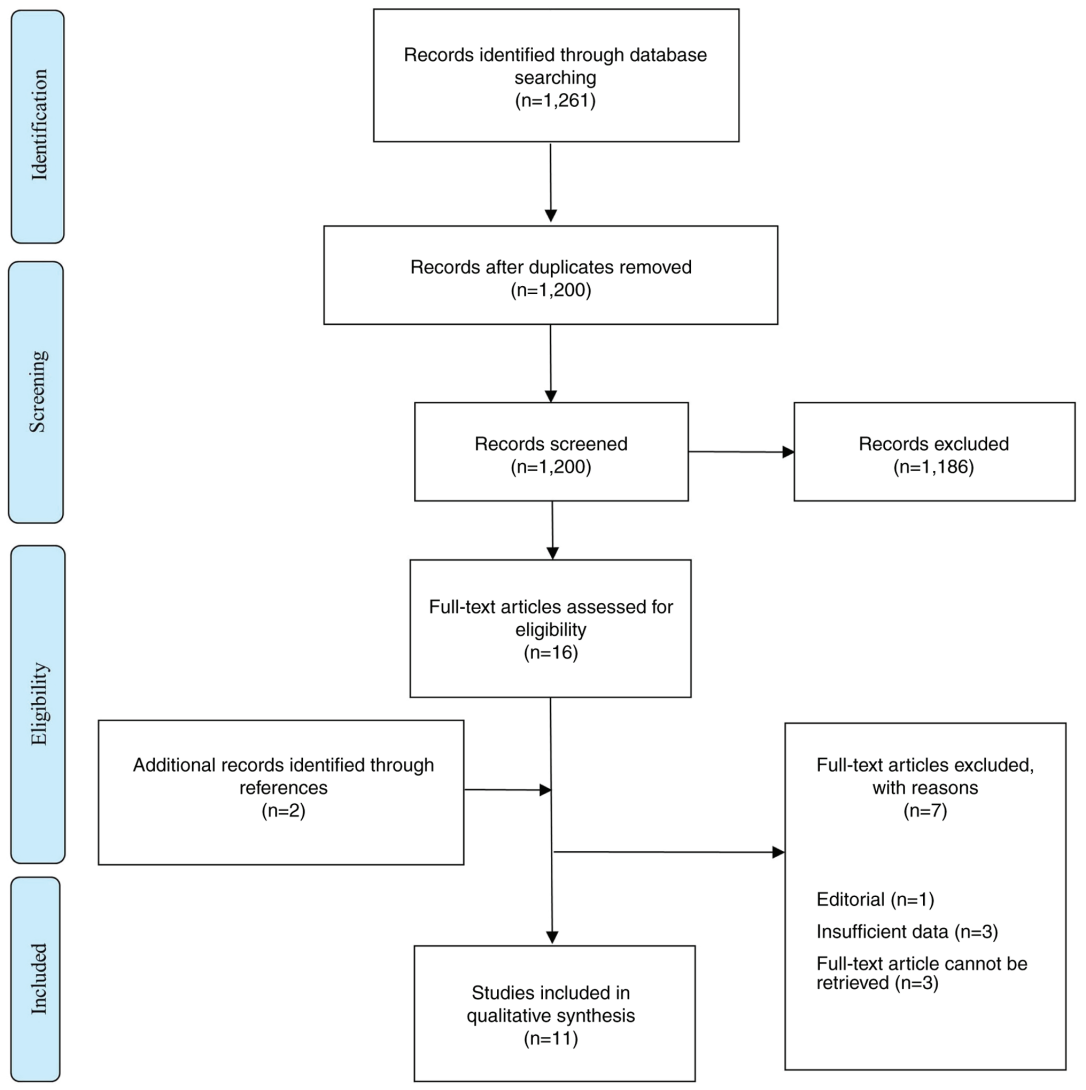
Preferred Reporting Items for Systematic Reviews and Meta-Analyses (PRISMA) flowchart (7) used for study selection.

A systematic search and narrative literature review were undertaken, compatible with the Preferred Reporting Items for Systematic Reviews and Meta-Analyses (PRISMA) guidelines (7), as presented in Fig. 1. A comprehensive electronic bibliographic search was performed in order to identify articles published from database inception to May 30, 2021. The electronic databases of PubMed, Scopus and ScienceDirect were searched using a combination of the following terms as either key words or Medical Subject Headings (MeSH): (awake) AND [(craniotomy) OR (microsurgery) OR (surgery)] AND [(arteriovenous malformations) OR (intracranial arteriovenous malformations) OR (brain arteriovenous malformations) OR (AVM)]. No limitations as to the year of publication, or study design were applied. The complete search strategy used for PubMed was as follows: (awake) AND [‘craniotomy’ (MeSH)] OR [‘microsurgery’ (MeSH)] OR (surgery) AND [‘Arteriovenous Malformations’ (MeSH)] OR [‘Intracranial Arteriovenous Malformations’ (MeSH)] OR (brain arteriovenous malformations) OR (AVM).

The titles and abstracts of all the retrieved articles were screened and examined to determine their relevance. Articles with titles and abstracts that met the inclusion criteria (as described below) were selected for further review. In addition, the references to retrieved full-text articles were examined to identify potentially relevant articles that were not detected through the electronic search.

### Study selection and quality assessment

Two impartial reviewers (AH and SC) screened THE titles and abstracts found through the initial search strategy to determine eligibility. In the case that the title and abstract were unclear regarding the inclusion of a study, the full text was obtained and evaluated to determine its eligibility. Differences in study eligibility were resolved by engaging in discussions with a third reviewer (GH) to achieve consensus when necessary.

For case reports, the quality of the included studies was evaluated using the Appraisal Checklist for case reports proposed by The Joanna Briggs Institute (JBI) (8). The tool consisted of eight questions to assess different domains of the study (clear reports of patient demographics, patient history, clinical condition, diagnostic/assessment tests, intervention/treatment, post-intervention clinical condition, adverse events and important takeaway lessons). Each question was answered with ‘yes’, ‘no’, ‘unclear’, or ‘not applicable’. One point was allocated for each ‘yes’ answer and the total score ranged between 0 and 8. The studies were categorized as follows: i) Score of 0 to 1 point, low quality; ii) score of 2 to 4 points, moderate quality; and iii) score of 5 to 8 points, high quality.

For case series, the quality of the included studies was evaluated using the Appraisal Checklist for case series proposed by The JBI (8). The tool consisted of ten questions to assess different domains of the study (clear reports of inclusion criteria, standardized method of measuring, valid methods for identification of all participants, consecutive inclusion of patients, complete inclusion of patients, patient demographics, clinical information, follow-up results, present sites/clinics demographic information and appropriate statistical analysis). Each question was answered with ‘yes’, ‘no’ or ‘unclear’. One point was allocated for each ‘yes’ answer and the total score ranged between 0 and 10. The studies were categorized as follows: i) Score of 0 to 3 points, low quality; ii) score of 4 to 6 points, moderate quality; or iii) score of 7 to 10 points, high quality.

For cohort studies, the quality of the included studies was evaluated using the Appraisal Checklist for cohort studies proposed by The JBI (8). The tool consisted of 11 questions to assess different domains of the study (study groups similarity, similar measurements of exposure, valid and reliable measure of exposure, identification of confounders, strategies to minimize confounders, all participants free of outcome at the start of the study, valid and reliable measure of outcomes, sufficient follow-up period, follow-up complete in all participants, strategies to address incomplete follow-up and appropriate statistical analysis). Each question was answered with ‘yes’, ‘no’ or ‘unclear’. One point was allocated for each ‘yes’ answer and the total score ranged between 0 and 11. The studies were categorized as follows: i) Scored of 0 to 4 points, low quality; ii) score of 5 to 7 points, moderate quality; or iii) score of 8 to 11 points, high quality.

### Eligibility criteria

The articles were eligible for the systematic review if they: i) Included adult patients; ii) the patients included underwent AC for AVM resection; iii) had any outcomes reported during and following AC; iv) were primary research articles; and v) were published in the English language. All review articles, commentaries and editorials were excluded.

### Outcomes of interest

The primary outcome of interest was to assess the risk-benefit of AC in AVM resection by recording all the neurological outcomes (complete resection status, intraoperative complications and post-operative neurological deficits). The secondary objective was to evaluate the disparity in the frequency of post-operative complications and follow-up complications between SMG I-II and SMG III-V.

### Data extraction

Using an extraction form in an Excel^®^ spreadsheet (Microsoft Corporation), AH and SC extracted the data. The data extracted included the following: Primary study author, year of publication, country, study design, population size undergoing AC, sex, mean age (in years), AVM location, hemorrhage or seizure at presentation, preoperative imaging, intraoperative neuromonitoring (IONM) method, SMG, follow-up duration (in months) and the aims of each study. Data on intraoperative complications, intraoperative airway difficulties or lack of cooperation, post-operative complications, postoperative neurological deficit, complete resection status and imaging outcomes were also extracted.

### Statistical analysis

Data processing and statistical analysis were stored and conducted on Microsoft Excel^®^ and R-Studio version 2024.4.0.735, respectively (9). Overall proportion with their 95% confidence intervals (CIs) were calculated for the categorical variables. The heterogeneity between the included studies was evaluated with I^2^ and P-values. In the case of P>0.05 and/or I^2^≤50%, the difference in heterogeneity between studies was considered statistically insignificant; hence, the meta-analysis was performed using a fixed-effects model. On the contrary, in the case of P≤0.05 and/or I^2^>50% the statistical heterogeneity between studies was considered significant, and the meta-analysis was performed using a random-effects model. Finally, when sufficient outcomes were available, subgroup analyses of proportions were conducted, regardless of heterogeneity, in studies with specific outcomes for SMG I-II and SMG III-V.

## Results

### Literature search results

Based on the search strategy, 1,261 titles and abstracts were retrieved (PubMed, 37; ScienceDirect, 1,181; Scopus, 43), and the subsequent screening process is presented in Fig. 1. Subsequently, 16 potentially relevant full-text articles were obtained for a detailed evaluation. In addition, two more potentially relevant articles were identified from the reference lists of the 16 full-text articles. Of note, four of the articles had missing information as regards the location, type, or treatment of the AVMs, and for another three articles, the retrieval of the full text was not achievable, despite multiple efforts being made; therefore, they were excluded. Of the 18 articles, 11 articles met the inclusion criteria and were selected for analysis in the present systematic review.

### Study and patient characteristics

A total of 11 studies with 106 patients who underwent AC were included in the present systematic review (1,4,10-18). More specifically, of the eligible studies included, six studies were conducted in the USA, two studies were conducted in China, and three studies were conducted in Japan, Pakistan, and Switzerland. The characteristics of the included studies are summarized in Table I. Out of the 11 studies, eight studies were case series (72.7%), two studies were case reports (18.2%), and one study was a cohort study (9.1%). Of note, nine studies reported follow-up durations, and the pooled mean follow-up duration was 18 months (range, 3-56.4 months). In addition, eight studies (72.7%) had a low risk of bias, while three studies (27.3%) had a moderate risk of bias (Table I).

As regards the patient characteristics (presented in Table II), it was found that among the 106 patients, 57 patients were male and 49 patients were female, with a mean age ranging from 23.5 to 67.1 years. Grade 3 AVMs were the most prevalent type of AVM, accounting for 35 cases (33.0%), and grade 2 AVMs accounted for 29 cases (27.4%), according to the preoperative analysis of the AVM and staging using the SMG. Preoperative imaging was performed in 99 patients (93.4%) and preoperative embolization was performed in 10 patients (9.4%). Only four studies (36.4%) reported the awake protocol employed, all of which used the asleep-awake-asleep technique.

### Intraoperative and post-operative findings

**Figure 2:**
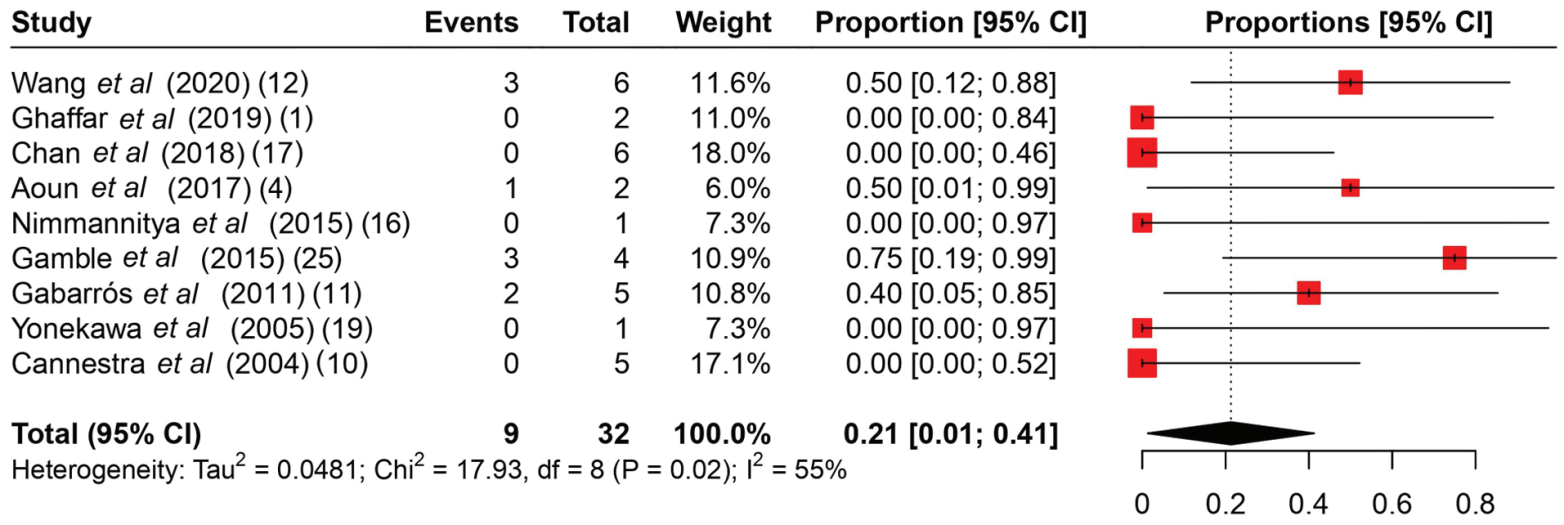
A proportional metanalysis of the rates of intraoperative complications. The studies analyzed are listed. The numbers in the parentheses indicate the year of publication and the relevant reference number. CI, confidence interval.

**Figure 3:**
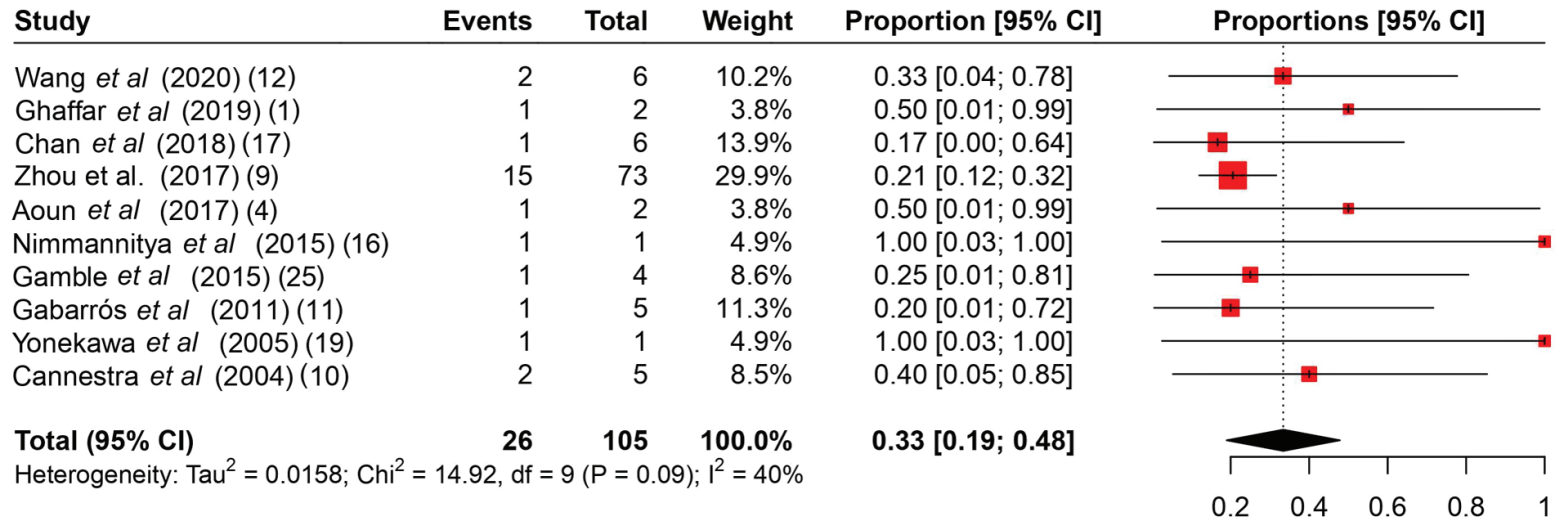
A proportional metanalysis of the rates of post-operative complications. The studies analyzed are listed. The numbers in the parentheses indicate the year of publication and the relevant reference number. CI, confidence interval.

**Figure 4:**
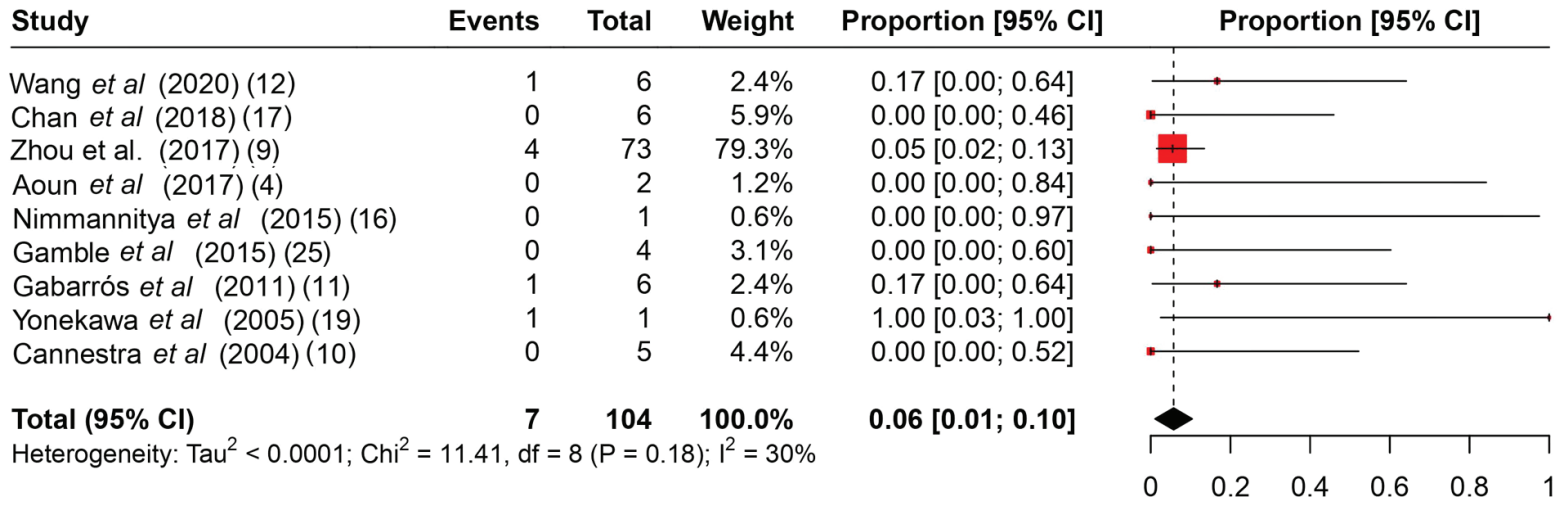
A proportional metanalysis of the rates of follow-up complications. The studies analyzed are listed. The numbers in the parentheses indicate the year of publication and the relevant reference number. CI, confidence interval.

Among the common intraoperative guidance methods (Table II), cortical stimulation, cortical mapping and continuous ECoG were the most frequently used methods. As regards intraoperative complications (Table III), it is evident that no major complications hindered the operations, and the majority were associated with intracranial hemorrhage during the operation or speech arrest, which was resolved gradually during follow-up. The rates of intraoperative complications ranged from 0 to 75%, with a summary estimate rate of 21% (95% CI, 1 to 41%; I^2^ 55%) (Fig. 2). Other post-operative complications (Table III) varied from mild facial weakness, aphasia and cranial nerve dysfunction to hemiplegia. The rates of post-operative complications ranged from 17 to 100% with a summary estimate rate of 33% (95% CI, 19 to 48%; I^2^ 40%) (Fig. 3). These complications were resolved in follow-up in almost all cases described. The rates of follow-up complications ranged from 0 to 100%, with a summary estimate rate of 6% (95% CI, 1 to 10%; I^2^ 30%) (Fig. 4).

### Resection status

**Figure 5:**
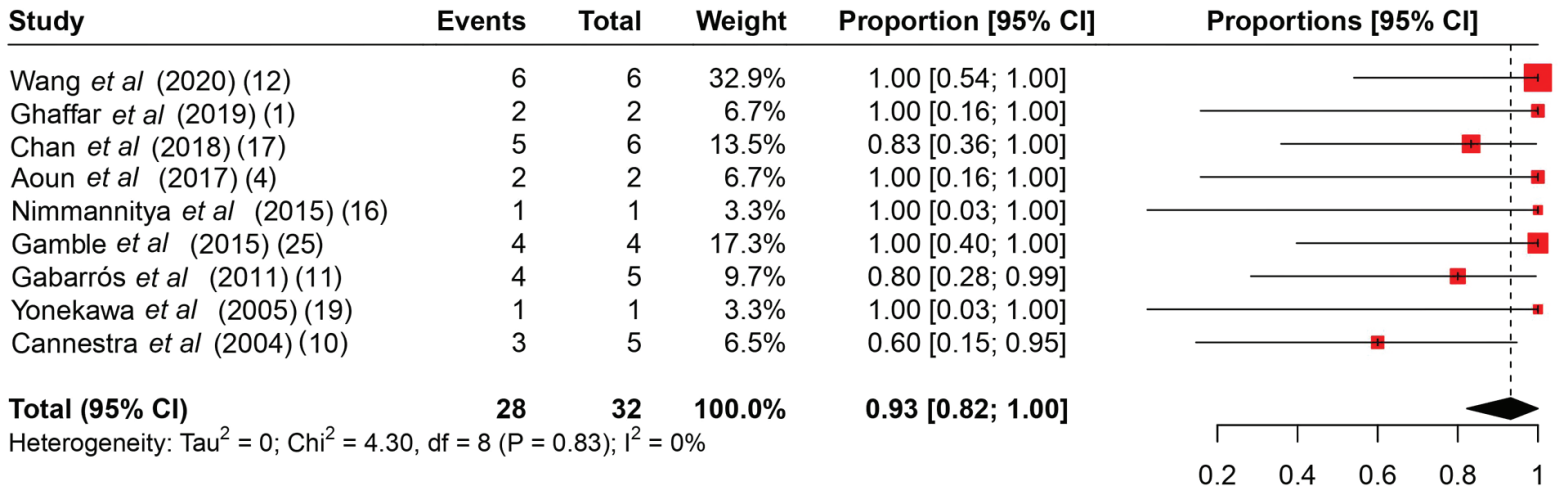
A proportional metanalysis of the rates of complete resection. The studies analyzed are listed. The numbers in the parentheses indicate the year of publication and the relevant reference number. CI, confidence interval.

The resection status of 74 patients (69.8%) was unreported (13,18). The rates of complete resection ranged from 60 to 100% with a summary estimate rate of 93% (95% CI, 82 to 100%; I^2^ 0%) (Fig. 5). Cannestra *et al* (18) included 2 patients who had AVMs that were deemed unresectable at the time of AC (no resection attempts were made), and they were referred for RS. Gabarrós *et al* (16) had 1 patient who could not achieve a complete resection and was also referred for RS. Wang *et al* (10) had 1 patient that required repeat AC to achieve complete resection.

### Subgroup analysis

**Figure 6:**
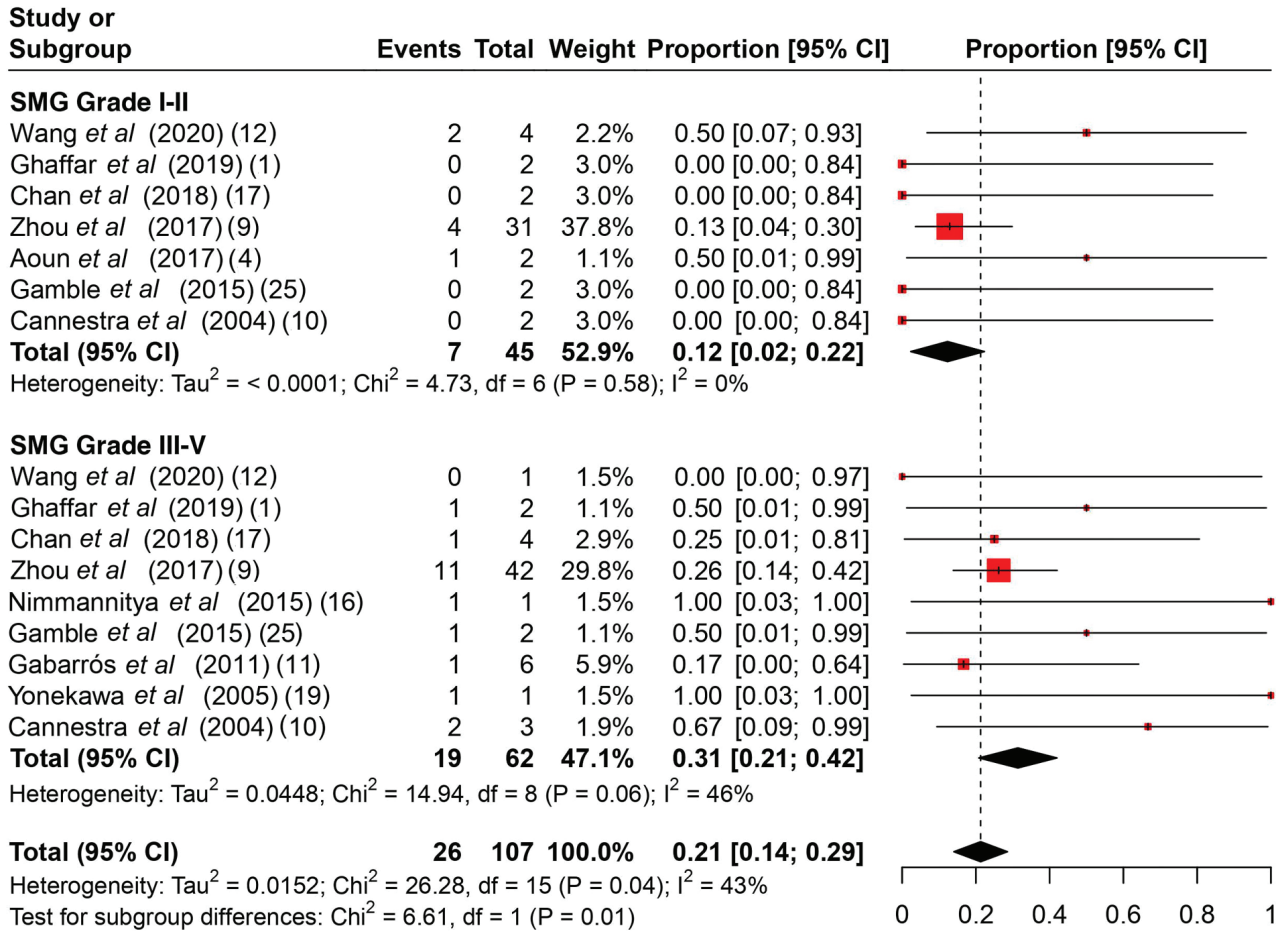
A subgroup proportional metanalysis of the rates of post-operative complications between SMG II and III-V. The studies analyzed are listed. The numbers in the parentheses indicate the year of publication and the relevant reference number. CI, confidence interval; SMG, Spetzler-Martin grade.

**Figure 7:**
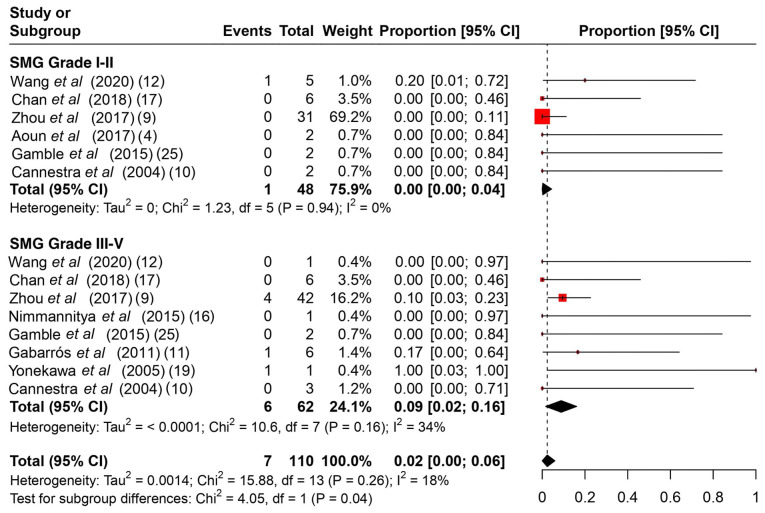
A subgroup proportional metanalysis of rates of follow-up complications between SMG II and III-V. The studies analyzed are listed. The numbers in the parentheses indicate the year of publication and the relevant reference number. CI, confidence interval; Spetzler-Martin grade.

The rates of post-operative complications among SMG I-II ranged from 0 to 50%, with a summary estimate rate of 12% (95% CI, 2 to 22%; I^2^ 0%) and among SMG III-V, these ranged from 0 to 100%, with a summary estimate rate of 31% (95% CI, 21 to 42%; I^2^ 46%) (Fig. 6). There was significant heterogeneity found between different dosage subgroups (P-value=0.01). The rates of follow-up complications among SMG I-II ranged from 0 to 20%, with a summary estimate rate of 0% (95% CI, 0 to 4%; I^2^ 0%) and among SMG III-V, these ranged from 0 to 100%, with a summary estimate rate of 9% (95% CI, 2 to 16%; I^2^ 34%) (Fig. 7). There was significant heterogeneity found between different dosage subgroups (P-value=0.04).

## Discussion

The significance of identifying the eloquent areas during brain surgery has been well-established, and currently, AC is the gold standard for identifying these regions during glioma resection (19). The present systematic review aimed to highlight the importance of awake craniotomy during AVM surgery and to identify the subgroups of patients that would benefit from such an intervention. Previously published data suggest that motor or language function may shift to an adjacent gyrus or to a contralateral hemisphere (20,21). This shift could occur due to brain plasticity in the presence of long-standing lesions, such as AVMs (20,21). Monitoring brain function and preventing mistargeting during AVM surgery is critical for optimizing outcomes (12). The precise identification and localization of the language function appear to be feasible only with the patient is in an awake condition (20,21). Additionally, it allows surgeons to determine the extent of the resection that can be performed safely without causing functional loss (14). The evaluation of the outcome following AVM surgery was performed using the modified Rankin Scale (mRS) (22). This scale assesses functional independence on a seven-grade scale. Chan *et al* (11) and Gabarrós *et al* (16) reported that 5 patients (83%) and 3 patients (60%), respectively, exhibited an improvement in their functional outcome after AC in terms of mRS.

Among the patients who experienced worsening immediately post-operatively, the majority of the patients had improved and only 6 patients had neurological deficits since the surgery. The rate of neurological deficit is higher compared with cases where AC is used for glioma surgery (~5.5%) (23); however, it should be noted that AC is a standard procedure in glioma surgery, while it was recently utilized in AVM surgery. Additionally, the data used in the present study were derived from different institutions with different methods of IONM and a lower number of patients, which may have affected the outcome.

A total of four studies included in the present systematic review revealed that immediate postoperative neurological deficits can arise despite an uneventful intraoperative course (1,11,14,17). Cerebral edema, vascular injury, or damage to areas with significant inputs to eloquent regions can all result in deficits (24). Although the majority of patients with post-operative deficits recover, a subset may suffer from a permanent loss of function. The predictors of long-term functional outcome include age, location, SMG, pre-operative deficits, surgical complications and new post-operative deficits (25). High-SMG AVM tends to have a more complicated post-operative and follow-up course (26). This was also reflected in the present study, as the majority of the patients who did have permanent neurologic deficits had a SMG ≥III. High-grade AVMs are large and can extend deep into the white matter, making surgical resection difficult and posing a greater risk for the post-operative neurological deficit (27). Therefore, one of the subgroups that can potentially benefit from AC are patients with an SMG #x003C;III. Further studies are required to determine whether AC is beneficial and safe for AVMs with SMG ≥III compared to other treatment modalities. It is also worth noting that all the studies included in the present systematic review had no reported mortality (1,4,10-18).

The majority of the patients (55.6%) presented with either seizure or hemorrhage, highlighting that AC was performed in individuals with ruptured AVM. This could target the second subgroup that can benefit from AC: Individuals with ruptured AVM (3,11). A previous randomized trial demonstrated that medical management was superior to interventional management (neurosurgical, radiosurgical or endovascular procedures) for unruptured AVMs in the short term (28). Of note, that trial ended early due to significant mortality in the surgical group (28,29). AC could potentially facilitate safer resection, and a new trial could be conducted using AC in the armamentarium for the surgical treatment of AVMs.

Currently, to the best of our knowledge, there are no publications available in the literature for AVM that directly compare AC with other treatment modalities. Extrapolating data from brain tumor resection surgery, AC is said to be economically advantageous from a cost and resource perspective due to the reduced need for post-operative intensive care and shorter hospital stays (23,30). It is important to acknowledge that there are substantial risks not to be underestimated when resecting AVM with AC. The main concerns are loss of airway patency, significant or unexpected hemorrhage, and hemodynamic instability (16). Mild hypoxemia can impair brain relaxation and surgical exposure (16). Controlling the airway could be challenging, particularly in a patient with rigid pin fixation (16). Other concerns include patient movement, which might jeopardize delicate microsurgical maneuvers and patient discomfort or fatigue during long procedures (4). It should be noted that the success of AC is largely dependent on anesthetic experience and patient cooperation (4). In the present systematic review, the patients in the included studies who had airway difficulties or were unable to co-operate received immediate anesthetic management and/or oxygen supplementation, aiding procedure completion. Sleep apnea was a prevalent risk factor among patients who encountered airway difficulties, which may have contributed to the airway obstruction experienced during AC (10). However, this was managed without the need for general anesthesia and subsided after inserting bilateral nasal airways (10). Of note, 1 patient experienced arterial desaturation during AC that was responsive to oxygenation via a standard face mask and persistent encouragement for the patient to continue deep breathing (13). However, it is important to note that this complication arose due to onyx migration from arterial embolization (13). Intraoperative anesthetic management should include vigilance for sudden hemodynamic changes that might require massive fluid resuscitation and pharmacological manipulation of the circulation (16).

AC has also maximized the extent of surgical resection. Surgeons can resect with more confidence in non-eloquent areas and avoid eloquent regions (31). Increasing the extent of resection can provide potential benefits, such as symptom relief and neurological improvement (32,33). The majority of the patients in the studies included in the present systematic review achieved complete resection. These findings suggest that AC could potentially be a safe procedure that can be undertaken for AVM resection, particularly in eloquent areas and in patients with a SMG I-II. The findings presented herein are consistent with those of a previous systematic review that indicated that AC may allow for the precise excision of eloquent AVMs, while preserving crucial brain functions (34). Additionally, significant flow reduction in AVM does not protect against hemorrhage, and treatment must continue until it is obliterated completely (16). Thus, complete resection is vital in providing protection from re-hemorrhaging, and AC allows for this by providing an opportunity to achieve maximal resection safely. Other forms of management, such as RS, do not provide this immediate protection (35,36). Endovascular embolization also plays a role in the management of cerebral AVM in eloquent areas (36). It has been employed as an adjuvant to surgery in the management of cerebral AVMs, particularly those >3 cm in size (10,37). However, endovascular embolization does carry specific risks. In the case report by Tolly *et al* (13), the patient first underwent onyx embolization followed by AC, and it was found that the patient later developed hypoxemia intraoperatively due to an onyx pulmonary artery embolism. Other complications of AVM embolization include intraoperative or post-operative hemorrhage, cerebral ischemia, transient or permanent neurologic deficit and mortality (38-40).

The limitations of the present systematic review include the small population size, limited data concerning follow-up, and marked conceptual heterogeneity among the included studies. This reduced the value of the interpretation of the statistical analysis conducted. The present systematic review has also highlighted the low number of studies currently available and the absence of studies directly comparing AC with asleep surgery (and other treatment modalities) in the resection of AVM. Thus, further studies comparing other treatment modalities with asleep microsurgery are warranted in order to determine its overall benefits, including the neurological outcome, length of hospital stay and effectiveness of the technique. It should also be highlighted that approximately half of the studies included herein had a follow-up period of ≤6 months. Therefore, any shortcomings observed during these periods could either be enduring or part of an ongoing developmental process. As a result, long-term follow-up studies are required to assess the impact of AC on AVM resection.

In conclusion, to date at least to the best of our knowledge, this is the first meta-analysis assessing the role of AC in AVM resection. The results suggest that AC could potentially be a useful tool in resecting AVMs, specifically in patients with eloquence involvement, SMG #x003C;3, and unruptured AVMs. However, the present systematic review was based on studies with marked heterogeneity in patient selection, AVM location, resection techniques, surgical team, equipment, field strength and follow-up information. Farther well-designed observational studies with longer follow-up periods are thus required to provide integrated information regarding the safety and benefits of AC in patients with AVM.

## Figures and Tables

**Table I:** Characteristics of the included studies.

Authors	Year of publication	Country	Study design	Population size for AC	Mean follow-up duration (months)	Risk of bias	(Refs.)
Wang *et al*	2020	USA	Case series	6	56.4	9/10	(10)
Ghaffar *et al*	2019	Pakistan	Case series	2	NR	5/10	(1)
Chan *et al*	2018	China	Case series	6	45	9/10	(11)
Zhou *et al*	2017	China	Cohort study	73	12	5/11	(12)
Tolly *et al*	2017	USA	Case report	1	3	8/8	(13)
Aoun *et al*	2017	USA	Case series	2	9^a^	9/10	(4)
Nimmannitya *et al*	2015	Japan	Case report	1	5	8/8	(14)
Gamble *et al*	2015	USA	Case series	4	NR	9/10	(15)
Gabarrós *et al*	2011	USA	Case series	5	25.9^a^	9/10	(16)
Yonekawa *et al*	2005	Switzerland	Case series	1	3	5/10	(17)
Cannestra *et al*	2004	USA	Case series	5	3	7/10	(18)

^a^Calculated from the data available in the studies. AC, awake craniotomy; NR, not reported.

**Table II:** Characteristics of the patients in the studies included in the present systematic review.

Authors, year of publication	Population size for awake surgery (M/F)	Mean age ± SD^a^ (years)	Hemorrhage or seizure at presentation (no. of patients)	AVM location (no. of patients)	Preoperative Embolization	Preoperative imaging^c^	SMG (no. of patients)	Awake protocol	IONM method	(Refs.)
Wang *et al*, 2020	6 (5M/1F)	67.1±17.2	Seizure (n=3)	Temporal (n=1), temporoparietal (n=1), frontal (n=2), frontoparietal (n=2)	2	6	Grade 2 (n=5), grade 3 (n=1)	AAA	Somatosensory-evoked potentials and DCS (language and motor functions). Continuous intracranial ECoG	(10)
Ghaffar *et al*, 2019	2 (F)	23.5±3.5	Seizure (n=1)	Frontal (n=1), temporoparietal (n=1)	1	2	Grade 1 (n=1), grade 3 (n=1)	NR	Cortical stimulation (verbal and motor response)	(1)
Chan *et al*, 2018	6 (3M/3F)	31.2±13.7	Hemorrhage (n=5), seizure (n=1)	Parietal (n=2), temporal (n=1), frontal (n=2), frontoparietal (n=1)	NR	NR	Grade 2 (n=2), grade 3 (n=4)	AAA	Cortical mapping (naming, comprehension, calculation and motor functions)	(11)
Zhou *et al*, 2017	73 (43M/30F)	34.9±17.5	Hemorrhage (n=41)	Eloquence involvement (n=24)	NR	73	Grade 1 (n=15), grade 2 (n=16), grade 3 (n=21), grade 4 (n=14), grade 5 (n=7)	NR	Neurophysiologic monitoring (brainstem auditory-evoked potential, electromyography, maximum expiratory pressure, somatosensory-evoked potential, visual-evoked potential, electroencephalography)	(12)
Tolly *et al*, 2017	1 (M)	26^b^	Seizure (n=1)	Supratentorial (n=1)	1	1	Grade 2 (n=1)	NR	NR	(13)
Aoun *et al*, 2017	2 (F)	46±10	Seizure (n=2)	Temporal (n=1), parietal (n=1)	NR	2	Grade 2 (n=2)	AAA	ECoG, intraoperative brain functional mapping, cortical mapping, and language/motor mapping	(4)
Nimmannitya *et al*, 2015	1 (F)	38^b^	Seizure (n=1)	Frontal (n=1)	1	1	Grade 3 (n=1)	AAA	Cortical mapping (motor and language), motor and visual naming tasks	(14)
Gamble *et al*, 2015	4 (1M/3F)	51.8±16.9	Hemorrhage (n=2), seizure (n=2)	Fronto-opercular (n=2), temporal (n=2)	NR	4	Grade 2 (n=2), grade 3 (n=2)	NR	Cortical mapping (language), subcortical stimulation	(15)
Gabarrós *et al*, 2011	5 (2M/3F)	31±9.7	Hemorrhage (n=2), seizure (n=3)	Frontoparietal (n=1), frontal (n=1), temporal (n=3)	4	5	Grade 3 (n=4), grade 4 (n=1)	NR	ECoG, somatosensory evoked potentials, language and motor mapping	(16)
Yonekawa *et al*, 2005	1 (F)	35^b^	NR	Parietal (n=1)	1	NR	Grade 4^a^	NR	NR	(17)
Cannestra *et al*, 2004	5 (2M/3F)	34.8±10.8	NR	Temporal (n=1), frontal (n=2), temporoparietal (n=2)	NR	5	Grade 2 (n=1), grade 2 to 1 (n=1), grade 3 (n=1), grade 3 to 2 (n=1), grade 4 to 3	NR	Language and motor mapping, continuous intracranial ECoG, intraoperative optical imaging of intrinsic signals	(18)

^a^Calculated from the data available in the studies;

^b^patient age (mean age could not be calculated as there was only 1 patient in the study);

^c^preoperative imaging included MRI and/or angiogram. M, male; F, female; NR, not reported; SD, standard deviation; AAA, asleep-awake-asleep DCS, direct cortical stimulation; AVM, arteriovenous malformation; SMG, Spetzler-Martin grade; IONM, intraoperative neuromonitoring; ECoG, electrocorticography.

**Table III:** Intra-operative and post-operative findings.

Authors, year of publication	Intraoperative complications (no. of patients)	Complete resection	Post-operative complications (no. of patients)	Follow-up neurological deficits (no. of patients)	Postoperative angiogram	(Refs.)
Wang *et al*, 2020	Seizures (n=1), blood transfusion (n=1), anxiety and nausea (n=1)	6/6	Hemorrhage requiring transfusion and mild facial weakness, which recovered in 3 days (n=1); left-sided weakness; recovered completely in 7 days (n=1)	Mild facial weakness, which recovered in 3 days (n=1); left-sided weakness; recovered completely in 7 days (n=1)	No residual malformation after 1st surgery (n=5); no residual malformation after 2nd surgery (n=1)	(10)
Ghaffar *et al*, 2019	None	2/2	Aphasia (n=1)	Aphasia resolved completely in 48 h (n=1).	No residual malformation	(1)
Chan *et al*, 2018	None	5/6	Hemorrhage with neurological deficit (n=1)	Improvement in functional outcome (n=5); no re-bleed or new neurological deficit (n=6)	NR	(11)
Zhou *et al*, 2017	NR	NR	Neurologic dysfunction (n=15)	Hemiplegia, cranial nerve dysfunction, hemianopia, or aphasia (n=4)	NR	(12)
Tolly *et al*, 2017	Hypoxemia^a^	NR	Onyx pulmonary arterial embolism (n=1)^a^	No new complaints (n=1)	NR	(13)
Aoun *et al*, 2017	Seizure (n=1)	2/2	Transient left facial droop and arm twitching (n=1)	No seizures, normal neurologic examinations and no speech impediments (n=2)	No residual malformation nor AV shunting	(4)
Nimmannitya *et al*, 2015	None	1/1	Speech difficulty (n=1)	Speech difficulty resolved (n=1); neuropsychological tests: No deterioration of cognitive or language functions (n=1)	No residual malformation	(14)
Gamble *et al*, 2015	Speech arrest (n=2); hemorrhage (n=1)	4/4	Dysphasia (n=1)	Dysphasia resolved (n=1)	No residual malformation	(15)
Gabarrós *et al*, 2011)	ICH (n=1); EDH (n=1)	4/5	Temporal seizure (n=1)	Temporal seizure continued persisting; improvement in functional outcome (n=3)	Residual malformation in one patient with incomplete resection	(16)
Yonekawa *et al*, 2005	None	1/1	Right-side hemiparesis appearing after 48 h (n=1)	Right side hemiparesis subsided almost completely (n=1)	No residual malformation	(17)
Cannestra *et al*, 2004	None (AVMs deemed unresectable, n=2)^b^	3/5	Word generation deficit at one month (n=2)	Word generation deficit resolved (n=1)	NR	(18)

^a^Complications were related to a prior procedure performed and not directly to awake craniotomy;

^b^AVMs deemed unresectable during awake craniotomy and thus resection was not attempted. NR, not reported; MINORS, methodological index for nonrandomized studies; AVMs, arteriovenous malformations; AV, arteriovenous ICH, intracerebral hematoma; EDH, epidural hematoma.

## Data Availability

The datasets used and/or analyzed during the current study are available from the corresponding author on reasonable request.

## References

[1] Ghaffar WB, Ahsan K, Shafiq F, Enam A (2019). Anaesthetic Management of Cerebral Arterio-venous malformation excision using Awake craniotomy: Initial experience of two cases. J Coll Physicians Surg Pak.

[2] Fotakopoulos G, Brotis AG, Fountas KN (2022). Dilemmas in managing coexisting arteriovenous and cavernous malformations: Case report. Brain Circ.

[3] Lawton MT, Rutledge WC, Kim H, Stapf C, Whitehead KJ, Li DY, Krings T, terBrugge K, Kondziolka D, Morgan MK (2015). Brain arteriovenous malformations. Nat Rev Dis Primers.

[4] Aoun RJN, Sattur MG, Krishna C, Gupta A, Welz ME, Nanney AD III, Koht AH, Tate MC, Noe KH, Sirven JI (2017). Awake surgery for brain vascular malformations and moyamoya disease. World Neurosurg.

[5] Solomon RA, Connolly ES Jr (2017). Arteriovenous malformations of the brain. N Engl J Med.

[6] Szelényi A, Bello L, Duffau H, Fava E, Feigl GC, Galanda M, Neuloh G, Signorelli F, Sala F (2010). Intraoperative electrical stimulation in awake craniotomy: Methodological aspects of current practice. Neurosurg Focus.

[7] Page MJ, McKenzie JE, Bossuyt PM, Boutron I, Hoffmann TC, Mulrow CD, Shamseer L, Tetzlaff JM, Akl EA, Brennan SE (2021). The PRISMA 2020 statement: An updated guideline for reporting systematic reviews. BMJ.

[8] Moola S, Munn Z, Tufanaru C, Aromataris E, Sears K, Sfetcu R, Currie M, Qureshi R, Mattis P, Lisy K, Mu PF https://synthesismanual.jbi.global.

[9] http://www.rstudio.com/.

[10] Wang AT, Pillai P, Guran E, Carter H, Minasian T, Lenart J, Vandse R (2020). Anesthetic management of awake craniotomy for resection of the language and motor cortex vascular malformations. World Neurosurg.

[11] Chan DYC, Chan DTM, Zhu CXL, Kan PKY, Ng AY, Hsieh YS, Abrigo J, Poon WS, Wong GKC (2018). Awake craniotomy for excision of arteriovenous malformations? A qualitative comparison study with stereotactic radiosurgery. J Clin Neurosci.

[12] Zhou Q, Li M, Yi L, He B, Li X, Jiang Y (2017). Intraoperative neuromonitoring during brain arteriovenous malformation microsurgeries and postoperative dysfunction: A retrospective follow-up study. Medicine (Baltimore).

[13] Tolly BT, Kosky JL, Koht A, Hemmer LB (2017). A case report of onyx pulmonary arterial embolism contributing to hypoxemia during Awake craniotomy for arteriovenous malformation resection. A A Case Rep.

[14] Nimmannitya P, Terakawa Y, Kawakami T, Tsuyuguchi N, Sato H, Kawashima T, Ohata K (2015). Awake craniotomy for cortical language mapping and resection of an arteriovenous malformation adjacent to eloquent areas under general anesthesia-A hybrid approach. Interdisciplinary Neurosur.

[15] Gamble AJ, Schaffer SG, Nardi DJ, Chalif DJ, Katz J, Dehdashti AR (2015). Awake craniotomy in arteriovenous malformation surgery: The usefulness of cortical and subcortical mapping of language function in selected patients. World Neurosurg.

[16] Gabarrós A, Young WL, McDermott MW, Lawton MT (2011). Language and motor mapping during resection of brain arteriovenous malformations: Indications, feasibility, and utility. Neurosurgery.

[17] Yonekawa Y, Imhof HG, Bjeljac M, Curcic M, Khan N

[18] Cannestra AF, Pouratian N, Forage J, Bookheimer SY, Martin NA, Toga AW (2004). Functional magnetic resonance imaging and optical imaging for dominant-hemisphere perisylvian arteriovenous malformations. Neurosurgery.

[19] Ibrahim GM, Bernstein M (2012). Awake craniotomy for supratentorial gliomas: Why, when and how?. CNS Oncol.

[20] Lazar RM, Marshall RS, Pile-Spellman J, Duong HC, Mohr JP, Young WL, Solomon RL, Perera GM, DeLaPaz RL (2000). Interhemispheric transfer of language in patients with left frontal cerebral arteriovenous malformation. Neuropsychologia.

[21] Lazar RM, Marshall RS, Pile-Spellman J, Hacein-Bey L, Young WL, Mohr JP, Stein BM (1997). Anterior translocation of language in patients with left cerebral arteriovenous malformation. Neurology.

[22] Pollock BE, Brown RD (2006). Use of the modified Rankin scale to assess outcome after arteriovenous malformation radiosurgery. Neurology.

[23] Serletis D, Bernstein M (2007). Prospective study of awake craniotomy used routinely and nonselectively for supratentorial tumors. J Neurosurg.

[24] Brown T, Shah AH, Bregy A, Shah NH, Thambuswamy M, Barbarite E, Fuhrman T, Komotar RJ (2013). Awake craniotomy for brain tumor resection: The rule rather than the exception?. J Neurosurg Anesthesiol.

[25] Gulati S, Jakola AS, Nerland US, Weber C, Solheim O (2011). The risk of getting worse: Surgically acquired deficits, perioperative complications, and functional outcomes after primary resection of glioblastoma. World Neurosurg.

[26] Chowdhury T, Zeiler FA, Singh GP, Hailu A, Loewen H, Schaller B, Cappellani RB, West M (2018). The Role of intraoperative MRI in awake neurosurgical procedures: A systematic review. Front Oncol.

[27] Derdeyn CP, Zipfel GJ, Albuquerque FC, Cooke DL, Feldmann E, Sheehan JP, Torner JC (2017). Management of brain Arteriovenous malformations: A scientific statement for healthcare professionals from the American Heart Association/American Stroke Association. Stroke.

[28] Mohr JP, Parides MK, Stapf C, Moquete E, Moy CS, Overbey JR, Al-Shahi Salman R, Vicaut E, Young WL, Houdart E (2014). Medical management with or without interventional therapy for unruptured brain arteriovenous malformations (ARUBA): A multicentre, non-blinded, randomised trial. Lancet.

[29] Magro E, Gentric JC, Darsaut TE, Ziegler D, Msi Bojanowski MW, Raymond J (2017). Responses to ARUBA: A systematic review and critical analysis for the design of future arteriovenous malformation trials. J Neurosurg.

[30] Zhang K, Gelb AW (2018). Awake craniotomy: Indications, benefits, and techniques. Colombian J Anesthesiol.

[31] Sacko O, Lauwers-Cances V, Brauge D, Sesay M, Brenner A, Roux FE (2011). Awake craniotomy vs surgery under general anesthesia for resection of supratentorial lesions. Neurosurgery.

[32] Englot DJ, Berger MS, Barbaro NM, Chang EF (2012). Factors associated with seizure freedom in the surgical resection of glioneuronal tumors. Epilepsia.

[33] Kim SS, McCutcheon IE, Suki D, Weinberg JS, Sawaya R, Lang FF, Ferson D, Heimberger AB, DeMonte F, Prabhu SS (2009). Awake craniotomy for brain tumors near eloquent cortex: Correlation of intraoperative cortical mapping with neurological outcomes in 309 consecutive patients. Neurosurgery.

[34] Rahman R, Majmundar N, San A, Sanmugananthan P, Berke C, Lang SS, Tayebi Meybodi A, Gajjar AA, Liu JK (2023). Surgical outcomes of awake craniotomy for treatment of arteriovenous malformations in eloquent cortex: A systematic review. World Neurosurgery.

[35] Friedman DI (2014). The pseudotumor cerebri syndrome. Neurol Clin.

[36] Pollock BE, Flickinger JC, Lunsford LD, Bissonette DJ, Kondziolka D (1996). Factors that predict the bleeding risk of cerebral arteriovenous malformations. Stroke.

[37] Senturk C

[38] Jayaraman MV, Marcellus ML, Hamilton S, Do HM, Campbell D, Chang SD, Steinberg GK, Marks MP (2008). Neurologic complications of arteriovenous malformation embolization using liquid embolic agents. AJNR Am J Neuroradiol.

[39] Sugiu K, Tokunaga K, Sasahara W, Watanabe K, Nishida A, Ono S, Nishio S, Date I, Rüfenacht DA (2004). Complications of embolization for cerebral arteriovenous malformations. Interv Neuroradiol.

[40] Lv X, Zhang Y, Wang J (2020). Systematic review of transcatheter arterial embolization of AVM: Indications, bleeding complications, cure rate, and long-term bleeding risk. Neurol India.

